# Clinical presentation and outcome in infantile Sandhoff disease: a case series of 25 patients from Iranian neurometabolic bioregistry with five novel mutations

**DOI:** 10.1186/s13023-018-0876-5

**Published:** 2018-08-03

**Authors:** Ali Reza Tavasoli, Nima Parvaneh, Mahmoud Reza Ashrafi, Zahra Rezaei, Johannes Zschocke, Parastoo Rostami

**Affiliations:** 1 Myelin Disorder Clinic (Iranian Neurometabolic Registery), Pediatric Neurology Division, Neurometabolic Registry Center, Children’s Medical Center, Tehran University of Medical Sciences, Tehran, Iran; 2 Division of Allergy and Clinical Immunology, Research Center for Immunodeficiencies, Children’s Medical Center, Tehran University of Medical Sciences, Tehran, Iran; 3 Division of Human Genetics, Medical University Innsbruck, Innsbruck, Austria; 4 Growth and Development Research Center, Division of Endocrinology and metabolism, Children’s Medical Center, Tehran University of Medical Sciences, Tehran, Iran

**Keywords:** Infantile Sandhoff disease, Organomegaly, Cherry red spot, HEXB gene

## Abstract

**Background:**

Infantile Sandhoff disease (ISD) is a GM2 gangliosidosis that is classified as a lysosomal storage disorder. The most common symptoms of affected individuals at presentation are neurologic involvement. Here we report clinical course and demographic features in a case series of infantile Sandhoff disease. Enzymatically and some genetically proven cases of ISD were extracted from the Iranian Neurometabolic Registry (INMR) in Children’s Medical Center, Iran, Tehran from December 2010 to December 2016.

**Result:**

Twenty five cases of infantile SD (13 female, 12 male) were included in this study. The age range of patients was 9–24 months with a mean of 15.8 months. The consanguinity rate of parents affected families was about 80%. The mean age of patients at disease onset was 6.4 months and the mean age at diagnosis was 14 months. Patients were diagnosed with a mean delay of 7.8 months. Eleven of patients died due to aspiration pneumonia and intractable seizure. The most common features at presentation (92%) were developmental delay or regression in speech and cognitive domains. Cherry red spots were detected in 17 patients (68%). Organomegaly was detected only in two patients. Enzyme studies showed marked reductions of both Hexosaminidase A and B in all patients. *HEXB* gene mutation studies performed in eight patients identified 6 different mutations, which five of them were novel.

**Conclusion:**

Infantile SD should be considered for each child presented with neurologic symptoms such as developmental delay and regression and cherry red spots in ophthalmic examination. Organomegaly is not a frequent clinical finding in infantile SD. Additionally; there are a genetic heterogenisity among Iranian patients.

## Background

Lysosomal storage disorders (LSDs) are a specific group of inborn errors of metabolism including more than fifthy different diseases caused by a structural defect or deficiency of lysosomal enzymes [1]. GM2 gangliosidosis is one group of LSDs that is classified into three types: Sandhoff (0 variant), Tay-Sachs (B variant) and GM2 activator deficiency (GM2A-AB variant). Mutations in *HEXA*, *HEXB* and *GM2A* genes that are inherited by autosomal recessive pattern lead to defective β-Hexosaminidase activity and accumulation of GM2 ganglioside in the intracellular organelles of visceral and neural cells [2]. Sandhoff disease (SD, OMIM 26880) due to a deficiency of β-Hexosaminidase activity (A and B units) was first described by Warren Tay in 1881 [3, 4]. The clinical manifestations of Tay-Sachs (OMIM 606869) and Sandhoff disease are the same and have been classified clinically into three forms of infantile, juvenile and adult. The clinical severity and age at disease onset are related to residual enzyme activity. Infantile SD presents with truncal hypotonia, muscle weakness, hyperacusis, developmental delay and regression, seizure and cherry red spots in ophthalmologic exam around 6 months of age. Hepatosplenomegaly, coarse facies and bone abnormality are less seen than Tay-Sachs disease. Death occurs by 3 years of age due to intractable seizure and aspiration pneumonia. The juvenile form of SD present between 2 and 10 years of age with dysarthria, ataxia, mental deterioration and seizures. Organomegaly and cherry red spots are uncommon. Adult form of SD is characterized by movement disorder, pyramidal and extra pyramidal signs and symptoms of lower motor neuron disease and supraneuclear ophtalmoplegia [2]. Molecular analysis and enzyme activity assessment are necessary to confirm the diagnosis of SD. There is no specific treatment for GM2 gangliosidosis but chaperone therapy with ketogenic diet and miglustat has been able to increase the cardiac function and reduce the frequency of seizures [3]. Due to the broad spectrum of clinical signs and symptoms of the disease and variety of mutations in the *HEXB* among the different races, herein we report the spectrum of clinical manifestations, clinical course and outcome of 25 cases of infantile SD presenting five novel mutations.

## Methods

**Fig. 1:**
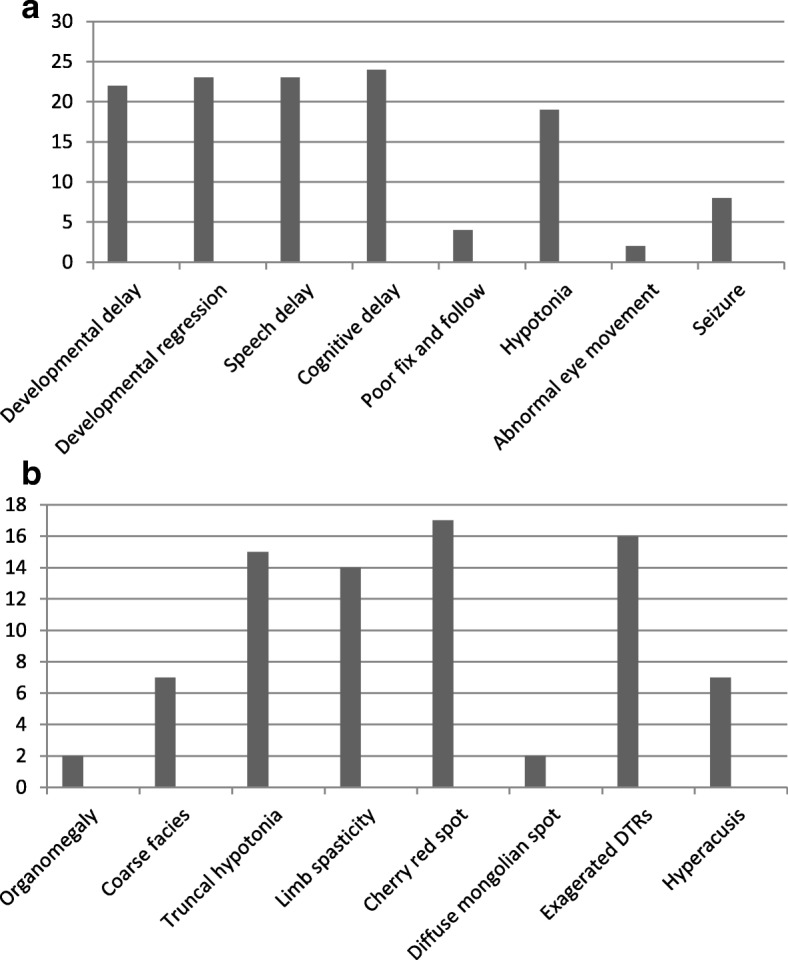
Step by step evaluation of a child suspected to infantile Sandhoff disease

We stablished a bioregistry system in our hospital Children’s Medical Center hospital, Tehran, Iran in 2010. During 7 years clinical and laboratory data of more than 270 patients with different types of neurometabolic disorders has been registered. Twenty five enzymatically and genetically proven cases of SD were extracted from the Iranian Neurometabolic Registry (INMR) from December 2010 to December 2017. Early diagnosis was carried out according to clinical manifestations, physical and neurologic examinations, neuro-imaging findings followed by assessment of hexosaminidase enzyme activity in peripheral blood leukocyte. The diagnosis of SD was confirmed by molecular study in some patients. All parents entered the study with informed consent (Fig. 1).

### β-Hexosaminidase activity assessment

The activity of β-Hexosaminidase was assessed through dried blood spots (DBSs) on filter paper for all included patients. The blood spots on filter paper placed in 3 mm diameter. After incubation of the samples at 37 °C, the amount of enzyme activity achieved by compared the value of hydrolyzed protein with a standard sample [5].

### Molecular study

Mutation analysis of *HEXB* gene was carried out on all coding exons and adjacent intron boundaries of the gene by PCR (Polymerase chain reaction) amplification and Sanger sequencing using standard methods.

## Result

### Demographic findings and clinical course

**Fig. 2:**
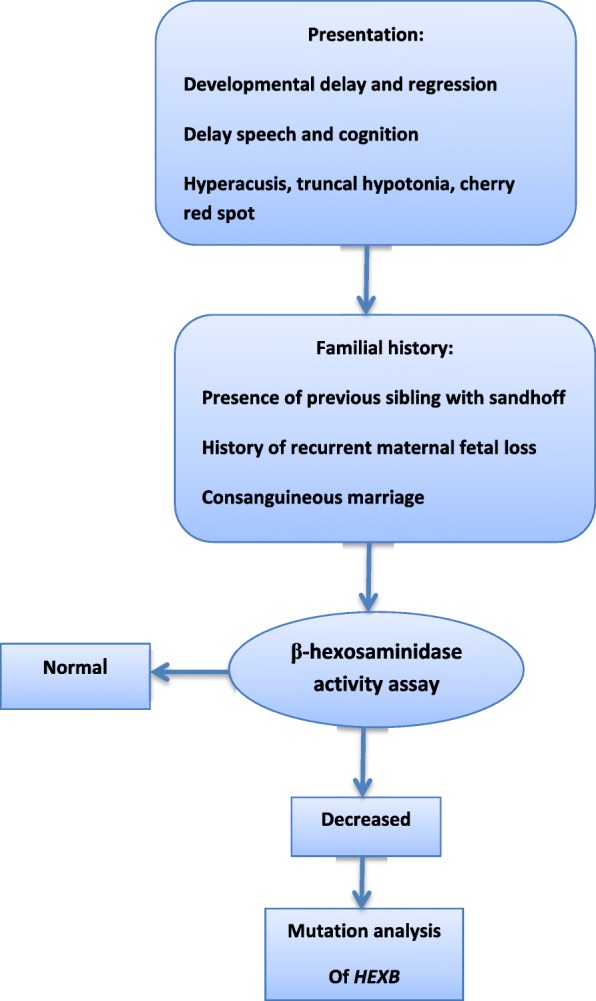
**a** Presenting clinical features of included patients; **b** physical examination of studied patients

Twenty five cases of infantile SD (13 female, 12 male) enrolled in the study. The age of patients was in the range of 9–24 months with mean age of 15.8 months. The consanguinity rate of parents was about 80%. Familial history of previous SD was revealed in three patients and nine mothers announced unexplained death in other sibling. In addition, two mothers had previous unexplained abortion. The mean age of patients at the onset of clinical manifestations was 6.4 months and the mean at diagnosis was 14 months. On average, patients were diagnosed with a mean delay of 7.8 months. Six patients had a history of prolonged neonatal jaundice. Six patients died due to aspiration pneumonia and intractable seizure (Fig. 2 and Table 1).

**Table 1:** Demographic features and family history of included patients

Patients NO	Age(m)	Age at (m) presentation	Age(m) diagnosis	Delay Of diagnosis (m)	Sex	Consanguineous Marriage	FH of Sandhoff Disease	History of previous disease	History of maternal abortion	FH of unexplained death	Dead/Alive
1	12	5	10	5	F	+	–	NI	–	–	Alive
2	16	10	11	1	F	+	+	NI	+	+	Alive
3	20	6	20	16	M	+	–	NI	–	+	Dead
4	18	7	18	11	M	+	–	–	–	–	Alive
5	10	3	10	7	M	–	–	–	–	–	Dead
6	12	7	11	4	F	+	–	–	–	–	Dead
7	20	9	19	10	F	–	–	–	–	–	Alive
8	20	12	20	8	M	+	–	–	–	–	Dead
9	9	5	9	4	F	+	–	–	–	–	Dead
10	22	6	22	16	M	+	–	–	–	–	Dead
11	20	6	18	12	F	+	–	–	–	–	Dead
12	18	6	18	12	F	+	–	–	–	–	Dead
13	14	2	12	10	F	+	–	NI	–	–	Alive
14	18	6	16	6	F	–	–	–	–	–	Dead
15	14	4	14	10	F	–	–	–	–	–	Alive
16	20	2	20	18	M	+	–	–	–	+	Alive
17	22	12	10	6	F	+	–	–	–	+	Alive
18	13	12	13	1	M	+	–	NI	–	–	Alive
19	12	5	12	7	M	+	–	–	+	+	Alive
20	11	9	11	2	M	+	–	NI	–	+	Dead
21	12	6	10	4	M	–	–	–	–	–	Alive
22	13	4	13	9	F	+	–	–	–	+	Alive
23	12	6	12	6	F	+	–	–	–	+	Alive
24	10	4	10	6	M	+	+	–	–	–	Alive
25	24	6	12	6	M	+	+	–	–	+	Dead

*M* month, *NL* normal, *NI* neonatal icterus, *FH* familial history, *F* female, *M* male, *NO* number

### Presenting symptoms

The most common clinical features of our patients at first presentation was developmental delay (92%) with a mean age at presentation of 4.9 months, regression in speech with a mean age at presentation of 10.7 month and cognitive decline with a mean age at presentation of 9.39 months. Hypotonia (mean age at presentation 5.2 months), seizure (mean age at presentation 5.6 months), poor fix and follow and abnormal eye movement (mean age at presentation 2.7 months) were less common symptoms in order of frequency (Fig. 2 and Table 2).

**Table 2:** Age of onset of clinical symptoms

Number of case	Age of developmental delay (m)	Age of developmental regression (m)	Age of Cognitive delay (m)	Age of poor fix and follow (m)	Age of hypotonia(m)	Age of seizure (m)	Age of delay speach
1	4	7	8	2	5	–	10
2	9	10	9	–	4	–	12
3	5	8	10	–	4	–	12
4	5	8	9	–	6	4	10
5	3	9	8	–	7	–	9
6	6	8	9	3	5	–	9
7	4	7	9	–	–	–	10
8	–	7	11	–	5	–	11
9	7	10	8	–	–	6	–
10	5	7	9	–	–	5	13
11	6	8	10	–	6	6	14
12	6	9	8	–	5	7	11
13	2	6	9	–	7	6	10
14	5	9	10	–	5	8	13
15	–	8	10	–	–	–	10
16	4	9	12	–	6	3	12
17	5	7	13	2	–	–	12
18	–	9	–	4	5	–	–
19	4	8	9	–	6	–	10
20	5	–	8	–	4	–	9
21	5	–	9	–	–	–	–
22	4	7	9	–	6	–	10
23	5	9	9	–	5	–	10
24	4	7	8	–	4	–	8
25	5	8	12	–	4	–	10

*M* month

### Clinical findings

On physical examination organomegaly, especially splenomegaly was detected only in two patients (by physical examination and then ultrasonography). Organomegaly was not developed in other patients in 1 year follow-up. The age of the onset of organomegaly was uncertain and this sign found out during first examination and 1 year follow-up of the patients. Seven of cases had coarse facies. The majority of patients had truncal hypotonia (64%) and lower limbs spasticity (56%). On ophthalmologic examination, cherry red spots were detected in 17 patients (68%), the remaining patients did not show this sign. Diffuse Mongolian spots were seen in two patients and seven of them (28%) had hyperacusis as an important clue for clinical diagnosis of GM2 Gangliosidosis. Exaggerated patellar and ankle tendon reflexes were detected in 64% of the patients (Fig. 2). During the study, 11 infants including 6 male and 5 females died of aspiration pneumonia and intractable seizure.

### Neuroimaging findings

The most common findings in brain on MRI (Magnetic Resonance Imaging) that carried out for ten patients were hypomyelination and/or delayed myelination, supratentorial brain atrophy, abnormal signals in basal ganglia especially putamen, globus pallidus and caudate nucleus (Table 3). Five series of brain MRI showed a radiologic sign as titled due to bilateral thalamus marked hyperintensity in T1-weighted images (thalamus brightness) and marked hypointensity in T2-weighted images of brain MRI.

**Table 3:** MRI findings in studied patients

Patient No	Brain MRI
5	Hypomyelination, caudate, GP, putamen involvement
9	Hypo and delayed myelination, putamen, GP and caudate involvement, Turkish mustache
10	Hypomyelination, putamen, GP and caudate involvement, Turkish mustache
11	Hypo and delayed myelination, putamen, GP and caudate involvement, Turkish mustache
12	Hypo and delayed myelination, putamen, GP and caudate involvement
14	Hypo and delayed myelination, putamen, GP and caudate involvement, Turkish mustache
16	Hypo and delayed myelination, demyelination, putamen, GP and caudate involvement, Turkish mustache
21	Hypomyelination, caudate, GP, putamen involvement
24	Hypomyelination, caudate, GP, putamen involvement
25	Hypomyelination, caudate, GP, putamen involvement

### Laboratory findings and *HEXB* mutations

The activity of hexosaminidase A, B, AB was low in all of patients. In eight families studied by mutation analysis, seven disease-causing mutations were found. Two mutations have been previously reported (c.1538 T > C; p.Leu513Pro and c.850 > T; p.Arg284Ter) [6, 7]. Five mutations were novel; they comprised two missense mutations (c.1602C > A; p.Cys534Ter and c.833C > T; p.Ala278Val), a six bp tandem duplication (c.668_669 + 4dupTGGTAA) and a 16 bp frameshift deletion (c.1642_1657delGCTGGATATTGTAACC) (Table 4). All the parents were heterozygote for *HEXB* gene mutations.

**Table 4:** Enzyme activity in plasma (nmol/ml/min) and result of mutation analysis

Patient No	Hexosaminidase A	Hexosaminidase B	Hexosaminidase AB	Mutations	Novel mutations
1	0.27	0.09	1.00	c.1602C > A (p.Cys534Ter)	+
2	0.18	0.00	0.5	c.833C > T (P.Ala278Val)	+
3	0.23	0.03	0.72	c.850C > T (p.Arg284Ter)	Zampieri 2009 [19]
4	0.12	0.78	0.46	c.1641_1657del	+
5	0.25	0.62	1.88	–	–
6	0.13	0.04	0.5	c.833C > T(p.Ala278val)	+
7	0.18	0.02	0.57	–	–
8	0.06	0.02	0.00	–	–
9	0.16	0.00	0.2	–	–
10	0.18	0.10	0.03	–	–
11	0.06	0.33	0.2	–	–
12	0.1	0.8	0.5		–
13	0.15	0.03	0.53	c.668_669 + 4dupTGGTAA	+
14	0.07	0.52	0.3	–	–
15	0.28	0.00	0.6	–	–
16	0.03	0.01	0.01	–	–
17	0.26	0.42	1	–	–
18	0.01	0.01	0.06	c.1538 T > c(rs778501777;p.leu513pro)	Lee et al. 2017 [7]
19	0.16	0.00	0.41	c.850 > T (p.Arg284Ter)	Zampieri 2009 [19]
20	0.16	0.00	0.67	–	–
21	0.21	0.03	0.98	–	–
22	0.08	0.00	0.3	–	–
23	0.20	0.10	0.6	–	–
24	0.04	0.19	0.00	–	–
25	0.23	0.00	0.8	–	–

Normal range of Hexosaminidase A: (0.96–1.78); Hexosaminidase B: (5.76–15.77); Hexosaminidase AB: (18.59–31.33)

## Discussion

SD constitutes 7% of GM2 gangliosidosis with an incidence of 1 in 384,000 live births that is associated with decrease of β-hexosaminidase activity [1]. In this study, we reviewed the most common presenting symptoms, clinical course and outcome of 25 cases of infantile-onset Sandhoff disease. Although the clinical features of infantile SD are non-specific in most patients, our experience showed motor and cognitive milestones delay and regression as the most common and the earliest features of the disease [2, 8]. The mean age at presentation in our cases of 6.4 months and a delay of diagnosis of 7.8 months were similar to other reports [2, 9]. As we previously mentioned clinical symptoms of the infantile SD, organomegaly was detected in only 2 patients over the time and follow-up of the patients for at least 1 year, so, unlike other GM2 gangliosidosis the lack of organomegaly could not preclude the presence of infantile SD [8, 10, 11]. Cherry-red spots are a characteristic ocular and physical sign of the infantile SD and can be used for early detection of suspected patients to this disease [2, 8]. In present study as other reports association between diffuse Mongolian spots with some lysosomal storage diseases and SD has been showed that may be a clue for diagnosis of lysosomal storage disorders including infantile SD [12, 13]. Accumulation of calcium associated with collection of GM2 ganglioside leads to gliosis and loss of myelin and axon in the cortical neurons. These evolutions give rise some earliest findings on T2-weighted images of brain MRI including bilateral thalamic hypotensity and hypomylination that are characteristic of brain involvement in the infantile SD disease which also were detected in our patients [14, 15]. SD is caused by mutation in *HEXB* gene is about 40 kb in length and located on 5q13 with 14 exons [6]. So far, 51 mutations have been identified in *HEXB*, the most of which are missense/nonsense (the human gene mutation database). Although there is no correlation between genotype and phenotype in SD but knowledge about mutations will facilitate genetic counseling and prenatal diagnosis in affected families [16]. Genetic study is important to make definitive diagnosis and to help family planning and prenatal diagnosis in the affected families. The presence of various and novel mutations in our patients may indicate heterogeneity in mutations in infantile SD among the Iranian population [17, 18]. We expect these mutations lead to partial or complete loss of protein, because the activity of HEXB enzyme were lesser than normal.

## Conclusion

Infantile SD should be considered for each child presented with neurologic regression especially during the first of life and presence of cherry red spots in ophthalmic examination. Absence of organomegaly cannot rule out the disease. Molecular analysis is important for definitive and prenatal diagnosis.

## Abbreviation

DBSs: dried blood spots
HEXB, A: Hexosaminidase A, B
INMR: Neurometabolic registry
ISD: Infantile Sandhoff disease
LSDs: Lysosomal storage disorders
MRI: Magnetic resonance imaging
PCR: Polymerase chain reaction
